# ATI-2173, a Novel Liver-Targeted Non-Chain-Terminating Nucleotide for Hepatitis B Virus Cure Regimens

**DOI:** 10.1128/AAC.00836-20

**Published:** 2020-08-20

**Authors:** Katherine E. Squires, Douglas L. Mayers, Gregory R. Bluemling, Alexander A. Kolykhalov, David B. Guthrie, Prabhakar Reddy, Debbie G. Mitchell, Manohar T. Saindane, Zachary M. Sticher, Vindhya Edpuganti, Abel De La Rosa

**Affiliations:** a Antios Therapeutics, Inc., Atlanta, Georgia, USA; b Emory Institute for Drug Development (EIDD), Atlanta, Georgia, USA; c Drug Innovation Ventures at Emory (DRIVE), Atlanta, Georgia, USA

**Keywords:** HBV DNA, antiviral agents, clevudine, hepatitis B virus, liver, nucleoside analogs, nucleotides, phosphoramidate, prodrug

## Abstract

ATI-2173 is a novel liver-targeted molecule designed to deliver the 5′-monophosphate of clevudine for the treatment of chronic hepatitis B infection. Unlike other nucleos(t)ides, the active clevudine-5′-triphosphate is a noncompetitive, non-chain-terminating inhibitor of hepatitis B virus (HBV) polymerase that delivers prolonged reduction of viremia in both a woodchuck HBV model and in humans for up to 6 months after cessation of treatment. However, long-term clevudine treatment was found to exhibit reversible skeletal myopathy in a small subset of patients and was subsequently discontinued from development. ATI-2173 was designed by modifying clevudine with a 5′-phosphoramidate to deliver the 5′-monophosphate to the liver. Bypassing the first phosphorylation step of clevudine, the 5′-monophosphate is converted to the active 5′-triphosphate in the liver. ATI-2173 is a selective inhibitor of HBV with an anti-HBV 50% effective concentration (EC_50_) of 1.31 nM in primary human hepatocytes, with minimal to no toxicity in hepatocytes, skeletal muscle, liver, kidney, bone marrow, and cardiomyocytes. ATI-2173 activity was decreased by viral polymerase mutations associated with entecavir, lamivudine, and adefovir resistance, but not capsid inhibitor resistance mutations. A single oral dose of ATI-2173 demonstrated 82% hepatic extraction, no food effect, and greatly reduced peripheral exposure of clevudine compared with equimolar oral dosing of clevudine. Despite reduced plasma clevudine exposure, liver concentrations of the 5′-triphosphate were equivalent following ATI-2173 versus clevudine administration. By selectively delivering the 5′-monophosphate to the liver, while retaining the unique anti-HBV activity of the 5′-triphosphate, ATI-2173 may provide an improved pharmacokinetic profile for clinical use, reducing systemic exposure of clevudine and potentially eliminating skeletal myopathy.

## Abstract (precis)

ATI-2173 is a novel liver-targeted molecule designed to deliver the 5′-monophosphate of clevudine for the treatment of chronic hepatitis B infection. Unlike other nucleos(t)ides, the active clevudine-5′-triphosphate is a noncompetitive, non-chain-terminating inhibitor of hepatitis B virus (HBV) polymerase that delivers prolonged reduction of viremia in both a woodchuck HBV model and in humans for up to 6 months after cessation of treatment. However, long-term clevudine treatment was found to exhibit reversible skeletal myopathy in a small subset of patients and was subsequently discontinued from development.

## INTRODUCTION

Hepatitis B virus (HBV) and subsequent chronic hepatitis B infection has a global impact on over 350 million individuals, predominantly impacting sub-Saharan Africa and East Asia (1). Left untreated, chronic HBV can lead to impaired liver function, cirrhosis, and eventually hepatocellular carcinoma (1). Accordingly, great effort has been invested into the development of treatments and, ultimately, a cure for HBV infection. Current options comprise interferon-α, which comes with myriad side effects, as well as chain-terminating nucleoside analogues, including entecavir, tenofovir, lamivudine, adefovir, and telbivudine (2). Although chain-terminating nucleoside analogues have a much-improved safety profile over interferon, they typically necessitate a longer duration of treatment that can result in viral breakthrough with drug resistance (3, 4) and have a low cure rate (5). Altogether, the landscape of HBV therapy is complex for patients and primed for the introduction of a curative treatment regimen (6).

Clevudine (l-FMAU; [1-(2-fluoro-5-methyl-β,l-arabinofuranosyl) uracil]), a small molecule inhibitor of HBV polymerase, was found to have antiviral activity in a woodchuck model of HBV infection nearly 20 years ago (7). Curiously, there was a dose-dependent delay in reemergence of viremia following withdrawal of treatment, with a select few animals remaining suppressed for months during the follow-up period, differentiating clevudine from the chain-terminating nucleoside analogues. In a subsequent phase IIA 28-day clinical trial, clevudine was found to be safe and effective (8). At the end of treatment, viral load (HBV DNA) was reduced by 2.5 to 3.0 log_10_, and viral suppression was sustained around a 2 log_10_ reduction from baseline for 6 months following treatment. Further, alanine aminotransferase (ALT) levels were reduced in all cohorts, with a subset of patients showing sustained normalized ALT at a 1-year follow-up.

The potent and prolonged HBV suppression (and associated biochemical response) was reproducible in multiple ensuing clinical studies, including 12-week treatment regimens (9, 10) and 24-week regimens in both e-antigen-positive (HBeAg^+^) (11) and -negative (HBeAg^−^) (12) patients. Despite the demonstrated safety of clevudine up to 24 weeks in the above-mentioned studies, reversible proximal myopathy symptoms emerged in South Korean patients after at least 8 months of clevudine exposure (13–15), prompting further investigation. Mitochondrial damage was not readily observed in the first report of clevudine-associated myopathy (14), and mitochondrial DNA polymerase γ was not found to be a target of clevudine in preclinical studies (16). However, a later report demonstrated long-term clevudine-associated myopathy was linked to mitochondrial injury by electron microscopy and mitochondrial DNA depletion (15), possibly due to exhaustion of available thymidine kinase 2 (TK2) (17), a key enzyme needed for mitochondrial DNA synthesis and utilized in the conversion of clevudine to the 5′-monophosphate.

**FIG 1:**
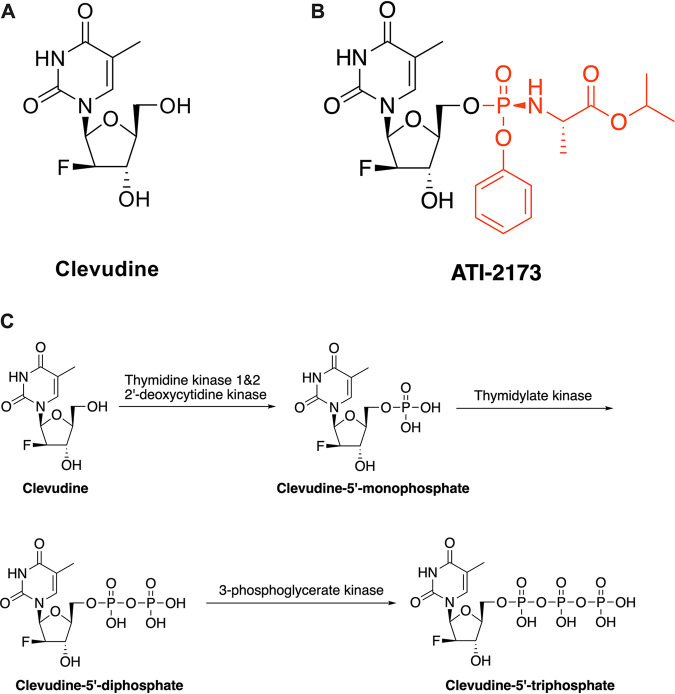
Structure and activation of clevudine and the novel prodrug ATI-2173. (A and B) ATI-2173 is structurally based on clevudine, with a phosphoramidate substitution at the 5′ hydroxyl group. (C) Clevudine is activated by three stepwise phosphorylations to form the clevudine-5′-triphosphate.

Clevudine (Fig. 1A), an unnatural l-nucleoside thymidine analogue (16), is unique as a noncompetitive, non-chain-terminating inhibitor of the HBV polymerase (18). Clevudine does so via a series of intracellular phosphorylations to generate the active 5′-triphosphate molecule (clevudine-5′-triphosphate) (16, 19) (Fig. 1B). ATI-2173 is a new chemical entity (patent no. 10,683,319, formerly EIDD-2173) derived from clevudine with a 5′-phosphoramidate modification (Fig. 1A) and the potential of reducing peripheral clevudine exposure by bypassing the first clevudine phosphorylation step, leading to ion trapping in the liver (20). Of note, any ATI-2173 that does enter peripheral circulation would not be a target for intracellular TK2, potentially mitigating the risk of enzymatic depletion and subsequent skeletal mitochondrial damage. We sought to define the anti-HBV biological and pharmacological properties of ATI-2173. Our results show that ATI-2173 is a highly selective and potent inhibitor of HBV replication *in vitro* and demonstrated a robust preclinical safety profile. Oral administration of ATI-2173 leads to a marked reduction in peripheral plasma clevudine while delivering the 5′-monophosphate to the liver. Altogether, ATI-2173, via the active 5′-triphosphate, is a potent non-chain-terminating anti-HBV nucleotide analogue that provides an improved pharmacokinetic profile over clevudine.

## RESULTS

### ATI-2173 is converted to the active 5′-triphosphate in hepatocytes.

**FIG 2:**
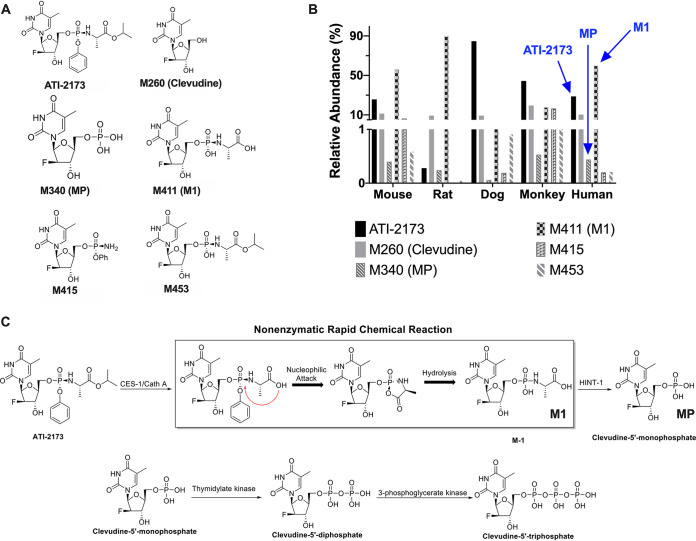
Proposed cellular metabolism of ATI-2173. (A) Structure of ATI-2173 and 5 metabolites found in hepatocytes of mouse, rat, dog, monkey, and human. (B) Relative abundance of each metabolite as a percentage of the total. In human hepatocytes, the parent compound (ATI-2173), major metabolite (M1), and clevudine-5′-monophosphate (MP) are indicated in blue. (C) ATI-2173 generates the major metabolite, M1, via CES-1/Cath A and a series of nonenzymatic rapid chemical reactions. M1 is then converted into MP putatively via HINT-1, where it follows normal clevudine metabolism to generate the active clevudine-5′-triphosphate.

ATI-2173 was incubated with cryopreserved mouse, rat, dog, monkey, and human hepatocytes for 4 h. Although ATI-2173 was not completely consumed, hepatocyte preparations from mice, rats, dogs, monkeys, and humans revealed five distinct metabolites, including clevudine and the 5′-monophosphate (MP) (Fig. 2A). The most abundant metabolite (other than the prodrug, clevudine, or MP) was M411 (M1), a product of rapid, nonenzymatic hydrolysis. ATI-2173 likely undergoes a preliminary hydrolysis step at the carboxyl ester catalyzed by either human cathepsin A (Cath A) and/or carboxylesterase 1 (CES-1), as is the case for other nucleos(t)ide phosphoramidates (20). This intermediate metabolite then undergoes rapid nonenzymatic nucleophilic attack on the phosphorous by the carboxyl group, leading to a short-lived intermediate that rapidly and noncatalytically hydrolyzes into the stable M1 metabolite. The relative abundance of each metabolite identified (Fig. 2B) indicates that ATI-2173 and M1 are the two most abundant species in human hepatocytes, followed by clevudine and MP, and finally two other minor metabolites (M415 and M453) that together account for less than 0.5% of the identified compounds. Following the formation of M1, the amino acid is cleaved to generate MP, putatively by the histidine triad nucleotide-binding protein 1 (HINT-1), a known bio-activator of pronucleotides (20–22). From here, the MP follows successive metabolism to generate the 5′-di- and 5′-triphosphates (23), which are not identified in the present study due to the relatively short incubation period. Figure 2C describes the proposed metabolites and associated metabolic enzymes. Sofosbuvir, an FDA-approved drug used in the treatment of HCV also possesses a 5′-phosphoramidate, undergoes a series of similar intracellular transformations, yielding an intermediate metabolite, before being converted to the 5′-monophosphate, 5′-diphosphate, and finally the active 5′-triphosphate (20). Though the backbone of ATI-2173 is an l-nucleotide, the metabolic pathway for releasing the 5′-monophosphate from the 5′-phosphoramidate in hepatocytes appears to follow a similar pathway to that of Sofosbuvir, a D-nucleotide.

### ATI-2173 is a potent and specific suppressor of HBV replication.

**FIG 3:**
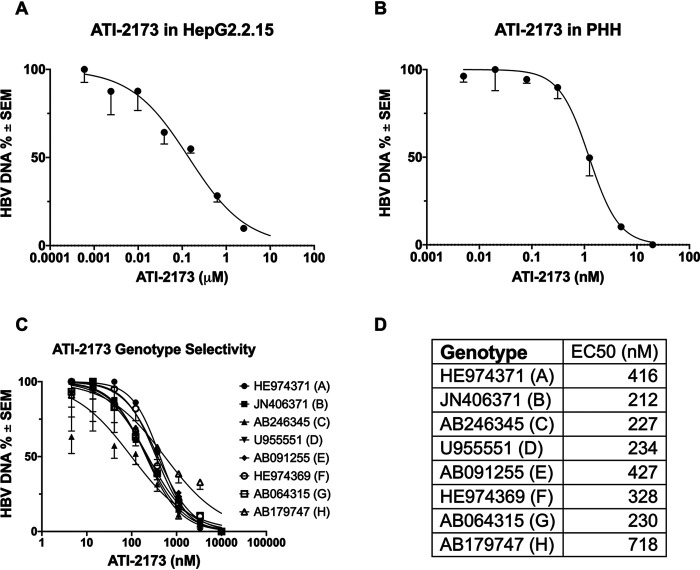
ATI-2173 is a potent and selective inhibitor of all HBV genotypes. (A) ATI-2173 inhibits HBV in HepG2 cells with an EC_50_ of 0.26 μM. (B) ATI-2173 inhibits HBV in primary human hepatocytes (PHHs) with an EC_50_ of 1.31 nM. (C) ATI-2173 is not genotype selective. (D) Summary of EC_50_ by genotype. GenBank IDs (left) correspond to the validated lab strains used in this assay.

Clevudine was found to have an anti-HBV EC_50_ of around 0.1 μM in cell-based *in vitro* systems (16, 24, 25). In humans, a dose found to be effective in the clinic (30 mg) corresponds with ∼1 μM plasma concentration and an intracellular 5′-triphosphate concentration of ∼10 μM (26) in primary human hepatocytes, although an EC_50_ value for clevudine in primary human hepatocytes has not been reported. As ATI-2173 is a liver-targeting 5′ phosphoramidate of clevudine, its active 5′-triphosphate would have similar *in vitro* activity to clevudine. ATI-2173 was incubated with HepG2.2.15 cells and primary human hepatocytes, and HBV DNA was quantified by quantitative PCR (qPCR). ATI-2173 was found to reduce extracellular HBV DNA in both cell types, with an EC_50_ of 0.26 μM in HepG2.2.15 cells (Fig. 3A; 50% cytotoxic concentration [CC_50_] >10 μM, Fig. S1) and an EC_50_ of 1.31 nM in primary human hepatocytes (PHHs) (Fig. 3B; CC_50_ >20 nM, Fig. S1). In these same assays, control compounds entecavir (EC_50_ of 0.6 nM, CC_50_ of >50 nM), clevudine (EC_50_ of 0.1 μm, CC_50_ of >10 μM), and peg-IFN-α2a (EC_50_ of 2 IU/ml, CC_50_ of >100 IU/ml) demonstrated potent and comparable anti-HBV activity (Fig. S2). In the same HepG2 assay, ATI-2173 showed similar activity across HBV genotypes A, B, C, D, E, F, G, and H (EC_50_ of 212 nM to 718 nM) (Fig. 3C and D). The EC_50_ variability is consistent with strain-to-strain differences, as seen with our positive-control compound, entecavir (Fig. S3). ATI-2173 was found to have no detectable efficacy up to 100 μM against a panel of viruses (hepatitis C virus, human immunodeficiency virus 1, influenza virus, respiratory syncytial virus, and herpes simplex virus 1) (data not shown). ATI-2173 had protein binding of approximately 55%, with no clear serum shift in EC_50_ (data not shown). Finally, when combined with other anti-HBV compounds in primary human hepatocytes, ATI-2173 had an anti-HBV activity that was additive with GLS4, lamivudine, and tenofovir (Table 1). Entecavir and adefovir had synergistic anti-HBV activity when combined with ATI-2173 (Table 1). Overall, these data indicate that ATI-2173 is an HBV-specific inhibitor of viral replication with nanomolar potency in primary hepatocytes and the potential for additive/synergistic activity when combined with other anti-HBV compounds.

**TABLE 1:** Combination effect of ATI-2173 plus other anti-HBV compounds on HBV activity in primary human hepatocytes.

Compound combined with ATI-2173	Combination index^*a*^	Combination effect
Entecavir	38.62	Minor synergism
GLS4	7.59	Additive
Lamivudine	−5.86	Additive
Tenofovir	8.56	Additive
Adefovir	32.85	Minor synergism

a A combination index of <25 is considered additive, while 25 to 50 is considered minor synergism.

### ATI-2173 is cross-resistant with other nucleosides.

Viral break-through, a phenomenon linked to resistance mutations in the HBV polymerase (27), has been demonstrated for chain-terminating nucleos(t)ides adefovir, lamivudine, telbivudine, and entecavir, among others (28–30). Although clevudine resistance and viral break-through was not found in 24-week, phase III clinical trials (11, 12), several patients did experience viral break-through associated with multiple mutations, predominantly M204I (4), after nine or more months of treatment. Notably, tenofovir and adefovir were active against these clevudine-resistant mutants, but not lamivudine nor entecavir (4). Since ATI-2173 and clevudine generate the same active 5′-triphosphate, it was hypothesized that ATI-2173 has the same resistance profile as that of clevudine. In HepG2 cells, ATI-2173 was incubated against ten mutant strains of HBV, eight of which confer nucleos(t)ide resistance (30–32), and two CpAM-resistant strains as non-nucleos(t)ide controls.

While ATI-2173 effectively inhibited replication of wild-type HBV, ATI-2173 resistance generally overlapped with lamivudine resistance, with the exception of M204V. The double and triple mutants associated with entecavir (and multidrug) resistance, rtS202G+M204I and rtS202G+M204I+M250V, also demonstrated cross-resistance with ATI-2173 (Table 2). As has been seen with other nucleosides (33, 34), the A181V mutation associated with adefovir resistance also generated resistance to ATI-2173. Based on the mechanism of action, the CpAM-resistant mutants, P25A and T33N, showed no cross-resistance with ATI-2173. Table 2 summarizes the cross-resistance found with ATI-2173 and entecavir, lamivudine, GLS4, or AT-130 resistance mutants.

**TABLE 2:** ATI-2173 cross-resistance^*a*^

Mutation	EC_50_ in μM				
	ATI-2173	Entecavir	Lamivudine	GLS4	AT-130
Wild type	0.601	0.003	0.096	0.015	0.246
rtM204I	>10	—	>10	—	—
rtV173L+M204I	>10	—	>10	—	—
rtM204V	0.162	—	3.509	—	—
rtL180M+M204V	>10	—	>10	—	—
rtS202G+M204I+M250V	>10	20.995	—	—	—
rtS202G+M204I	>10	9.962	—	—	—
rtA181V	>10	—	0.210	—	—
rtN236T	0.386	—	0.130	—	—
coreP25A	0.537	—	—	0.295	—
coreT33N	0.338	—	—	3.081	>10

a ATI-2173 was tested against eight nucleoside-resistant HBV genotypes and two cpAM-resistant HBV genotypes. Symbol —, not applicable.

### ATI-2173 has no apparent toxic effect on cells.

**FIG 4:**
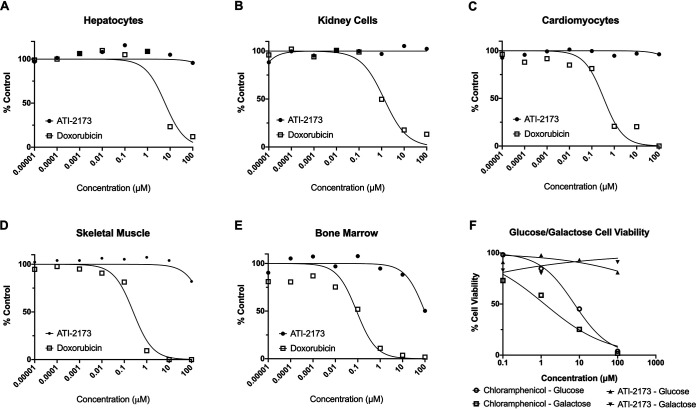
ATI-2173 is nontoxic at clinically relevant concentrations to hepatocytes, kidney cells, cardiomyocytes, skeletal muscle cells, bone marrow, and mitochondria. ATI-2173 was exposed for 48 to 72 h to hepatocytes (A), kidney cells (B), and cardiomyocytes (C), and was found to exhibit no observable cytotoxicity. Doxorubicin was used as a control. ATI-2173 was exposed for 48 to 72 h to skeletal muscle cells (D) and bone marrow cells (E) and was found to exhibit very mild (D) or mild (E) cytotoxicity at 100 μM doses. (F) ATI-2173 was incubated with HepG2 cells in medium supplemented with either 20 mM glucose or 10 mM galactose. While the chloramphenicol (positive control)-treated cells saw increased cell death in galactose-treated medium compared to glucose-treated medium, cells treated with ATI-2173 saw no appreciable cell death in either glucose or galactose medium.

ATI-2173 was evaluated for potential acute cellular and mitochondrial toxicity. Proliferating cells (HK-2 human kidney cells and SkMC human skeletal muscle cells) and nonproliferating cells (cryopreserved primary human hepatocytes, cryopreserved iCell cardiomyocytes, and cryopreserved human bone marrow mononuclear cells) were incubated with increasing concentrations of ATI-2173 or the control compound doxorubicin. ATI-2173 up to 100 μM did not demonstrate any cytotoxicity in primary hepatocytes, kidney cells, or cardiomyocytes (Fig. 4A to C). Minimal cytotoxicity was observed for bone marrow cells and skeletal muscle cells at concentrations of 100 μM or greater, (Fig. 4D and E), which is >100-fold higher than relevant clinical doses. Similar to ATI-2173 and consistent with previous literature (35), clevudine did not demonstrate cytotoxicity, except minimally in bone marrow cells (CC_50_ of >100 μM) (Fig. S4).

The glucose/galactose assay was conducted in HepG2 cells with increasing concentrations of ATI-2173 and the control compound chloramphenicol to assess potential mitochondrial toxicity. Although cell toxicity increased with chloramphenicol (due to mitochondrial toxicity), ATI-2173 demonstrated no apparent toxicity, with no differences in cell viability in glucose-supplemented medium versus galactose-supplemented medium (Fig. 4F), indicating no apparent mitochondrial toxicity. Similarly, clevudine demonstrated no differences between glucose and galactose cell viability, indicating no apparent mitochondrial toxicity (Fig. S4). Altogether, these data suggest ATI-2173 has a low risk of acute cytotoxicity and mitochondrial toxicity at doses relevant to the clinic.

### ATI-2173 is orally bioavailable and liver targeted.

**FIG 5:**
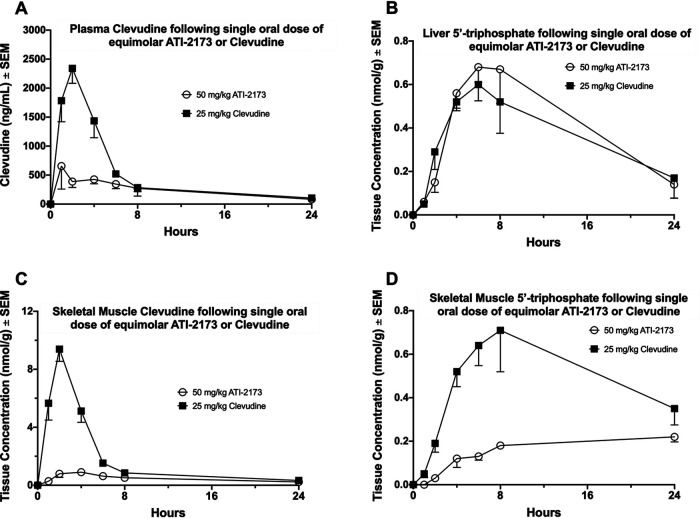
Plasma analyte concentration from rats dosed with equimolar single oral doses of ATI-2173 (50 mg/kg) or clevudine (25 mg/kg). (A) Plasma was isolated over 24 h and assessed for unphosphorylated clevudine. Tissues were collected at different time points following dosing and assessed for 5′-tripohosphate in the liver (B), unphosphorylated clevudine in skeletal muscle (C), or 5′-triphosphate in skeletal muscle (D).

Clevudine is orally bioavailable and yielded plasma concentrations up to 1 μM at doses found to be both effective (8, 10) but also linked with skeletal myopathy (15). Ideally, an improved pharmacokinetic profile would demonstrate a significantly reduced plasma exposure of clevudine following ATI-2173 dosing, while maintaining similar liver exposure of the 5′-triphosphate. Sprague-Dawley rats were given equimolar, single oral doses of either ATI-2173 (50 mg/kg) or clevudine (25 mg/kg), and plasma samples were collected at 1, 2, 4, 6, 8, and 24 h postdose. Figure 5A shows a nearly 5-fold reduction in peak plasma concentration of clevudine following ATI-2173 administration compared to clevudine administration at an equimolar dose. Clevudine and 5′-triphosphate levels in liver and skeletal muscle tissue samples from rats dosed as described above were measured at 1, 2, 4, 6, 8, and 24 h postdose. Figure 5B demonstrates nearly identical liver exposure to the 5′-triphosphate following equimolar dosing of ATI-2173 and clevudine, despite considerable reduction in plasma exposure to clevudine with ATI-2173 dosing (Fig. 5A). Accordingly, skeletal muscle concentrations of clevudine (Fig. 5C) and the 5′-triphosphate (Fig. 5D) showed a nearly 10-fold and 4-fold reduction in peak concentrations, respectively, following administration of ATI-2173 versus clevudine. These results are consistent with the marked reduction in plasma exposure to clevudine. Rats administered a single oral 50 mg/kg ATI-2173 dose had no discernible differences in plasma exposure (maximum concentration of drug in serum [C_max_] or area under the concentration curve from 0 to last [AUC_0-last_]) to ATI-2173 or clevudine (data not shown) when fed or fasted. ATI-2173 underwent high hepatic extraction (82%) in cynomolgus monkeys with portal vein cannulation following single oral (20 mg/kg) and intravenous (2 mg/kg) administration (data not shown). These results indicate ATI-2173 is both orally bioavailable and liver targeted, which leads to reduced systemic plasma and skeletal muscle clevudine exposure while still maintaining effective concentrations of active 5′-triphosphate in the liver.

## DISCUSSION

The active 5′-triphosphate of clevudine has a unique mechanism of action as a noncompetitive, non-chain-terminating inhibitor of the HBV reverse transcriptase (18). It binds to and distorts the active site of the polymerase such that it blocks DNA replication at all stages (priming initiation, priming polymerization, and DNA synthesis), but is not incorporated into the elongating chain, unlike traditional chain-terminating nucleos(t)ide analogues. The 5′-triphosphate is also unique from other nucleos(t)ide analogues in that as few as 4 weeks of daily administration can maintain suppressed viremia for months following treatment cessation in the woodchuck model of HBV (7) and in chronically infected HBV patients (8). ATI-2173 is a novel 5′-phosphoramidate of clevudine, which inhibits HBV with nanomolar potency in primary human hepatocytes. No significant acute, off-target mitochondrial or cellular toxicities were detected *in vitro* at clinically relevant concentrations. Sequence divergence among HBV populations throughout the world has led to different genotype classifications. In general, genotypes A and D predominate in central and southern Europe (36), genotype D prevails in the middle east (37–39), genotypes B and C dominate Asia (40, 41), and genotypes E to H are less common or more globally restricted (42). ATI-2173 demonstrates similar activity across all genotypes.

Similar to chain-terminating nucleos(t)ide analogues, the active 5′-tripohosphate of ATI-2173 is susceptible to a number of resistance mutations and long-term monotherapy may be challenging with viral breakthrough and drug resistance (4). Mutations associated with clevudine resistance in the clinic, such as M204I, were also linked with ATI-2173 resistance and overlap lamivudine and entecavir mutations. Overall, ATI-2173 resistance overlaps with lamivudine and entecavir. A181V, an adefovir-associated resistance mutation, was also found to reduce ATI-2173 anti-HBV EC_50_. Combination therapy in the clinic of 20 mg clevudine and adefovir effectively maintained viral suppression at 96 weeks without generating resistance (43). In primary human hepatocytes, ATI-2173 demonstrated additive-to-synergistic anti-HBV activity in combination with adefovir, entecavir, and tenofovir, indicating combination therapy may lead to improved treatment or cure regimens. Though cross-resistance and viral reemergence could be of clinical concern for ATI-2173, chain-terminating nucleos(t)ide analogues with a higher barrier to resistance, such as tenofovir (44), could be advantageously paired with ATI-2173 to target multiple mechanisms of DNA replication, preventing viral breakthrough and emergence of HBV drug resistance, and result in a potent combination to potentially shut down the HBV polymerase activity.

ATI-2173 targets the 5′-monophosphate to the liver and leads to an improved pharmacokinetic safety profile. Monkeys dosed with ATI-2173 showed an 82% hepatic extraction ratio, while rats dosed with ATI-2173 had significant reductions in plasma clevudine, skeletal muscle clevudine, and skeletal muscle 5′-triphosphate exposures compared to rats dosed with equimolar clevudine. Despite significant reductions in peripheral clevudine, liver concentrations of the 5′-triphosphate remained nearly equivalent between ATI-2173- and clevudine-treated rats, indicating ATI-2173 may maintain the same benefits of the active 5′-triphosphate in the liver while minimizing the risks associated with peripheral clevudine exposure. The 5′-phosphoramidate of ATI-2173 generates the 5′-monophosphate bypassing the first phosphorylation step of clevudine, which uses the TK2 enzyme and may be responsible for the reversible skeletal myopathy seen in chronically dosed patients (15, 17, 45). Thus, ATI-2173 that does leak into the systemic circulation may be less likely than clevudine to lead to myopathy. Altogether, ATI-2173 is a novel 5′-phosphoramidate of clevudine that delivers the 5′-monophosphate to the liver, reducing peripheral exposure to clevudine and potentially reducing the risk of skeletal myopathy. A drug candidate such as ATI-2173 that maintains 5′-triphosphate (a nucleotide analogue with a unique mechanism of action) levels in the liver but decreases plasma nucleoside exposure may have an improved clinical profile, particularly when paired with a chain-terminating nucleos(t)ide analogue that has a different mechanism of action and different bioactivation pathway (e.g., tenofovir) for chronically infected HBV patients (18). To this end, a phase I clinical trial is ongoing to investigate the safety, tolerability, pharmacokinetics, and anti-HBV activity of ATI-2173 in healthy subjects and subjects with chronic HBV (46).

## MATERIALS AND METHODS

### Metabolite identification.

ATI-2173, clevudine, clevudine-5′-triphosphate, and reference standards were provided by WuXi SynTheAll. Positive-control reference standards were obtained from Toronto Research Chemicals: 7’-ethoxycoumarin (7-EC), 7-hydroxycoumarin (7-HC), 7-hydroxycoumarin β-D-glucuronide (7-HCG), and 7-hydroxycoumarin sulfate (7-HCS). Cryopreserved hepatocytes from male CD-1 mice, Sprague-Dawley rats, beagle dogs, cynomolgus monkeys (pooled from *n* ≥3), and human hepatocytes (single donor) were obtained from BioIVT (Baltimore, MD). InVitroGRO HT medium and Kreb’s Henseleit buffer were purchased from BioIVT. On the day of incubation, hepatocytes were thawed in a water bath at 37°C, resuspended in prewarmed InVitroGRO HT, centrifuged (50 × *g* for 5 min at room temperature), and the pellet resuspended in prewarmed Kreb’s Henseleit buffer. Cell densities were adjusted to 2 × 10^6^ viable cells/ml using Kreb’s buffer. Hepatocytes were incubated with 10 μM ATI-2173 (or 100 μM 7-EC as a positive control) at 37°C and 5% CO_2_ for 0 or 4 h. Incubations were quenched with ice-cold acetonitrile:methanol (9:1 vol/vol) solution, vortexed, and centrifuged at 10,000 rpm at 4°C for 10 min. The supernatants were concentrated and reconstituted in methanol:water (1:1 vol/vol), centrifuged at 10,000 rpm at 4°C for 10 min, and the supernatant subjected to liquid chromatography-mass spectrometry (LC/MS) analysis. Samples were analyzed by ultraperformance liquid chromatography (UPLC)/UV (Thermo Vanquish Flex UHPLC with Vanquish Quaternary Pump F, Vanquish Split Sampler FT, and Vanquish Column Compartment, Thermo Vanquish Variable Wavelength Detector, Phenomenex Luna C18, 3 μm, 150 × 4.6 mm at 30°C). The mobile phases were 0.1% formic acid in water and 0.1% formic acid in acetonitrile. Metabolites were coded with the mono-isotopic mass (e.g., M411 has mass of 411 Da.) and relative abundance (single calculation) was semiquantitatively determined as a percentage of all metabolites plus unchanged drug.

### Anti-HBV activity in HepG2.2.15 cells.

ATI-2173 was obtained from WuXi SynTheAll. Entecavir (positive control) was obtained from Shanghai Titan Scientific Co., Ltd. HepG2.2.15 cells (derived from human hepatoblastoma cell-like HepG2) contain four 5′ to 3′ tandem copies of the HBV genotype D and were provided by WuXi. HepG2.2.15 cells were cultured in Dulbecco’s modified Eagle medium (DMEM)/F12 (Invitrogen, 11330032) supplemented with 2% fetal bovine serum (FBS) (HyClone, SV30087.03), 1% l-glutamine, 1% minimum essential medium (MEM) nonessential amino acid solution (NEAA), and 1% penicillin-streptomycin solution. QIAamp 96 DNA blood kit was purchased from Qiagen (51162). On day 1, HepG2.2.15 cells were seeded in a 96-well plate at a density of 6.0 × 10^4^ cells/well and cultured at 37°C and 5% CO_2_. Culture medium containing ATI-2173 or entecavir (serially diluted) was added to cells on day 2 and refreshed on day 5. ATI-2173 dilutions were: 0.0006, 0.0024, 0.0098, 0.0391, 0.1563, 0.6250, 2.5000, and 10.0000 μM. On day 8, cell culture supernatants were collected and DNA was extracted by the QIAamp 96 DNA blood kit. qPCR was run at 95°C for 10 min, then cycled at 95°C for 15 s, 60°C for 1 min, for a total of 40 cycles. HBV DNA content in the samples was calculated according to the standard curve and C*_T_* (cycle threshold of qPCR) values of a sample. Data were normalized for maximum and minimum copy numbers.

### Anti-HBV activity across different genotypes and resistance mutations in HepG2 cells.

ATI-2173 was obtained from WuXi SynTheAll. HepG2 cells were purchased from ATCC (HB-8065) and cultured in RPMI 1640 (Gibco, A10491-01) supplemented with 10% FBS (HyClone, SV30087.03), 2 mM glutamine, 100 U/ml penicillin, and 100 μg/ml streptomycin. HepG2 cells were transiently transfected with HBV DNA plasmids (Fugene HD) on day 1 and transfected cells were seeded into a 96-well plate at a density of 1.5 × 10^4^ cells/well. Genotype plasmids were as follows: genotype A2, pcDNA3.1-HE974371-1.1mer (GenBank ID HE974371); genotype B, pcDNA3.1-JN406371-1.1mer (GenBank ID JN406371); genotype C, pcDNA3.1-AB246345-1.1mer (GenBank ID AB246345); genotype D, pcDNA3.1HBV-wt (GenBank ID U95551); genotype E, pcDNA3.1-AB091255-1.1mer (GenBank ID AB091255); genotype F2, pcDNA3.1-HE974369-1.1mer (GenBank ID HE974369); genotype G, pcDNA3.1-AB064315-1.1mer (GenBank ID AB064315); and genotype H, pcDNA3.1-AB179747-1.1mer (GenBank ID AB179747). Resistance plasmids were based on wild-type U95551 and included rtM204I, rtV173L+M204I, rtM204V, rtL180M+M204V, rtS202G+M204I+M250V, rtS202G+M204I, rtA181V, rtN236T, P25A, and T33N. ATI-2173 serial dilutions were 4.5725, 13.7174, 41.1523, 123.4568, 370.3704, 1111.1111, 3333.3333, and 10,000.0000 nM. ATI-2173 was incubated with HepG2 cells as described above for HepG2.2.15 cells. On day 8, cells were lysed with 0.33% NP-40 and centrifuged. The supernatants were collected and treated with DNase at 37°C for 30 min, then 75°C for 15 min. DNA was finally extracted with DNA extract solution and incubated at 65°C for 6 min, then 98°C for 2 min. DNA was quantified as described above.

### Anti-HBV activity in primary human hepatocytes.

ATI-2173 was obtained from WuXi SynTheAll. Pegylated interferon-α2a (positive control) was obtained from Roche. Primary human hepatocytes (cryopreserved, single donor, lot no. QBU) were provided by WuXi and cultured in DMEM (Gibco, 1965-092) supplemented with 10% FBS (HyClone, SV3008703) and 1% penicillin/streptomycin (Invitrogen, SV30010). HBV genotype D was concentrated from HepDE19 culture supernatants (2.53 × 10^10^ genome equivalents [GE]/ml) and provided by WuXi. On day 0, hepatocytes were seeded into a 48-well plate at a density of 1.32 × 10^5^ cells/well. On day 1, hepatocytes were infected with 200 HBV GE/cell and were cultured at 37°C and 5% CO_2_. On day 2, ATI-2173 or peg-IFN-α2a was serially diluted with dimethyl sulfoxide (DMSO) and added to culture medium (final concentration of DMSO in medium was 2%). ATI-2173 dilutions were 0.005, 0.020, 0.078, 0.313, 1.250, 5.000, and 20.000 nM. On day 8, the culture supernatants were collected and HBV DNA was harvested with QIAamp 96 DNA blood kit (Qiagen, 51162). DNA was quantified by qPCR as described above.

For HBV combination studies, primary human hepatocytes were treated as described above and single-compound assays were conducted to determine the appropriate test concentrations. ATI-2173 was tested at concentrations between 0.5 nM and 8 nM. Entecavir was obtained from Shanghai Titan Scientific Co., Ltd. and tested at concentrations between 0.0025 nM and 0.04 nM. GLS4 was obtained from WuXi AppTec and tested at concentrations between 50 nM and 800 nM. Lamivudine was obtained from Dalian Meilun Biotech Co., Ltd. and tested at concentrations between 2.5 nM and 40 nM. Tenofovir DF was obtained from Shanghai Titan Scientific Co., Ltd. and tested at concentrations between 0.125 nM and 2 nM. Adefovir dipivoxil was obtained from Sun Chemical Technology (Shanghai) Co., Ltd. and tested at concentrations between 0.25 nM and 4 nM. Combination indexes were determined using MacSynergy and the 3-D model described by Prichard and Shipman (47). Combination indexes less than 25 were considered additive, between 25 and 50 were considered minor synergism, between 50 and 100 were considered moderate synergism, and greater than 100 were considered strong synergism.

### Cytotoxicity.

HK-2 human kidney cells were obtained from ATCC (Manassas, VA) and cultured in Gibco Keratinocyte serum-free medium (Thermo Fisher Scientific, Waltham, MA) supplemented with 10% FBS (Peak Serum, Wellington, CO). Human skeletal muscle cells were obtained from Lonza (Walkersville, MD) and cultured in SkGM-2 skeletal muscle cell growth medium-2 (Lonza). Both HK-2 and skeletal muscle cells were cultured humidified at 37°C and 5% CO_2_. Cryopreserved human primary hepatocytes and medium were received from Lonza. Cryopreserved iCell cardiomyocytes and cardiomyocyte medium were purchased from Stem Cell Technologies (Vancouver, BC, Canada). Cryopreserved human bone marrow mononuclear cells were purchased from Lonza with HemaTox Myeloid medium purchased from STEMCELL Technologies (Vancouver, BC, Canada). All cryopreserved cells (human primary hepatocytes, iCell cardiomyocytes, and human bone marrow mononuclear cells) were stored in vapor phase liquid nitrogen until the day of plating. ATI-2173 was obtained from WuXi and doxorubicin was purchased from Sigma Millipore (Burlington, MA). Serial dilutions of compounds in DMSO were as follows: 100, 10, 1, 0.1, 0.01, 0.001, 0.0001, and 0.00001 μM (final concentration of DMSO in medium was 0.5%).

HK-2 and skeletal muscle cells were seeded into 96-well plates at 5,000 cells/well and incubated overnight at 37°C and 5% CO_2_. Human hepatocytes were thawed, diluted in hepatocyte plating medium (Lonza) and seeded into a 96-well, collagen-coated plates at 2.5 × 10^4^ cells/well. Hepatocytes were incubated for 6 h at 37°C in 5% CO_2_, then the medium was aspirated and replaced with hepatocyte culture medium at 50 μl/well, and finally incubated overnight. The iCell cardiomyocytes were thawed, suspended in iCell thawing/plating medium, and plated at 4 × 10^4^ cells/well in a gelatin-coated 96-well plate. iCells were incubated for 2 days at 37°C in 7% CO_2_. After 2 days of incubation, iCells were visually checked to ensure a uniform layer of attached cells. Human bone marrow cells were thawed, diluted in HemaTox medium, and seeded into a 96-well tissue culture plate at 5 × 10^4^ cells/well. Bone marrow cells were incubated for 1 h at 37°C in 5% CO_2_.

Following incubation, cells were dosed and incubated for 72 h at 37°C and 5% CO_2_. Following incubation, the endpoint cell viability CellTiter-Glo 2.0 assay (Promega, San Luis Obispo, CA) was performed and luminescence data collected on a Synergy 4 plate reader (Biotek, Winooski, VT). Where possible, the 50% inhibitory concentration (IC_50_) was calculated using the Biotek GEN5 software.

### Mitochondrial toxicity.

Proliferating human hepatocytes (HepG2 cells) were acquired from Southern Research Institute. ATI-2173 was provided by WuXi and chloramphenicol was purchased from Millipore Sigma. Cells were cultured in 225 cm^2^ tissue culture flasks (Corning) containing 50 ml of DMEM (Life Technologies), 20 mM glucose (Millipore Sigma, St. Louis, MO), and 10% FBS (termed “glucose medium”). At least 1 week prior to seeding in assay plates, the HepG2 cultures were split into parallel cultures, where one culture continued in glucose medium while the second culture was grown in DMEM containing 10 mM galactose (Sigma Millipore) and 10% FBS (“galactose medium”). Cells were incubated at 37°C with 5% CO_2_. On day 1, HepG2 cells were harvested from the glucose and galactose media parallel cultures seeded on 6-well plates with 1.5 × 10^5^ cells/well in the appropriate glucose or galactose medium. Following seeding, cells were dosed with controls and compounds to give in-well concentrations of 100, 10, 1, and 0.1 μM (final concentration of DMSO was 0.5%). Cells were subcultured at a 1:3 ratio, and redosed on days 4 and 8. On day 11, cells were harvested and viable cell counts were determined by hemocytometer with trypan blue as the viability indicator.

**Ethics statement.** All animal protocols using rats were reviewed and approved by the Institutional Animal Care and Use Committees (IACUC) at the facilities where the studies were performed. Animal experiments were conducted in the Association for Assessment and Accreditation of Laboratory Care (AAALAC)-accredited and Public Health Services Animal Welfare Assurance-approved animal care suites at the Yerkes National Non-Human Primate research facility.

### ATI-2173 pharmacokinetics and tissue concentration.

Female Sprague Dawley (SD) rats (from Envigo, NJ), 225 to 249 g, were used in the studies. Clevudine and ATI-2173 were administered by oral gavage (p.o.) in carboxyl methyl cellulose/PEG 400/Tween 20 (0.5%/20%/5%). The oral doses were administered at 25 mg/kg and 50 mg/kg, respectively. Blood samples were collected at 1, 2, 4, 6, 8, and 24 h into lithium-heparin microtainer tubes (BD). Plasma samples were prepared within 30 min after collection by centrifugation at 2,000× rcf for 10 min at 4°C and stored at –80°C before processing for analysis by liquid chromatography-tandem mass spectrometry (LC-MS/MS). Rat organs (skeletal muscle, liver, and heart) were collected from all rats immediately following blood collection at all time points. The tissues were immediately snap-frozen in liquid nitrogen and stored at –80°C before processing for analysis by LC-MS/MS.

Aliquots of rat plasma were precipitated with acetonitrile and centrifuged at 1,500 rcf for 10 min at 4°C. The supernatants were transferred to an HPLC vial for analysis by LC-MS/MS. Frozen rat tissue samples (∼50 mg) were extracted with 70% acetonitrile in water at 4°C and homogenized in a Bead Ruptor 24 (Omni International, Kennesaw, GA, USA). To remove large solids, homogenates were centrifuged at 1,500× rcf for 10 min at 4°C. The clarified supernatants were transferred to microcentrifuge tubes and centrifuged again at 15,000× rcf for 10 min at 4°C. The remaining supernatant was transferred to an HPLC vial for analysis by LC-MS/MS.

Samples were maintained at 4°C in a Leap Pal Autosampler (CTC Analytics AG, Zwingen, Switzerland). Plasma concentrations of ATI-2173 and clevudine were determined using qualified methods with an assay range of 10 to 1,000 ng/ml. Calibrators and quality control samples were prepared in blank rat plasma (Pel Freez Biologicals, Rodgers, AR, USA) and showed linearity with *R*^2^ values of >0.99. QCs were prepared at 30, 500, and 900 ng/ml and were measured within ± 30% of actual values. HPLC analysis of ATI-2173 was performed with an Agilent 1200 HPLC (Agilent Technologies, Santa, Clara, CA, USA) equipped with a column oven, UV lamp, and binary pump. For ATI-2173, a 5-min isocratic HPLC method with 0.1% formic acid in HPLC-grade water and methanol (45:55) was employed with an ACE 5 C_18_ column (50 × 2.1 mm, 5 μm particle size) (Advanced Chromatography Technologies LTD., Aberdeen, Scotland). Mass spectrometry was performed on an API 4000 mass spectrometer (Sciex, Framingham, MA, USA) using positive electrospray ionization (ESI) in multiple reaction monitoring mode (MRM). For clevudine and its internal standard, a 6-min gradient HPLC method was used from 15% mobile phase B to 90% mobile phase B with a Hypercarb PGC column (100 × 4.6 mm, 5 μm particle size) (Thermo Fisher Scientific, Waltham, MA, USA). Mobile phase A consisted of 25 mM ammonium bicarbonate in HPLC grade water at pH 9.8 and acetonitrile was used as mobile phase B. Mass spectrometry was performed on a QTRAP 5500 mass spectrometer (Sciex, Framingham, MA, USA) using positive electrospray ionization (ESI) in multiple reaction monitoring mode (MRM). Data analysis was performed using Analyst Software (AB Sciex, Framingham, MA, USA).

Tissue levels were measured according to qualified LC-MS/MS methods with an assay range of 18 to 18,000 ng/g of tissue. Calibrators were prepared in blank rat tissue lysate and showed linearity with *R*^2^ values of >0.99. HPLC separation was performed on an Agilent 1200 system (Agilent Technologies, Santa Clara, CA, USA) equipped with a column oven, UV lamp, and binary pump. For the separation of ATI-2173 and its internal standard, an Acclaim Polar Advantage II column (150 × 4.6 mm, 5 μm particle size) (Thermo Fisher Scientific, Waltham, MA, USA) was used. A 4-min gradient from 5% to 60% mobile phase B was used with 50 mM ammonium formate in HPLC-grade water as mobile phase A and acetonitrile as mobile phase B. Mass spectrometry was performed on a QTRAP 5500 mass spectrometer (Sciex, Framingham, MA, USA) using positive electrospray ionization (ESI) in multiple reaction monitoring mode (MRM). For the separation of clevudine, clevudine-5′-monophosphate, and clevudine-5′-triphosphate and internal standards, a Hypercarb PGC column was used (100 × 4.6 mm, 5 μm particle size) (Thermo Fisher Scientific, Waltham, MA, USA). A 7-min gradient from 5% to 90% mobile phase B was used with 25 mM ammonium bicarbonate in HPLC-grade water at pH 9.8 as mobile phase A and acetonitrile as mobile phase B. Mass spectrometry was performed on a QTRAP 5500 mass spectrometer (Sciex, Framingham, MA, USA) using negative electrospray ionization (ESI) in multiple reaction monitoring mode (MRM).

## Supplementary Material

Supplemental file 1
