# Supplementary material for: ATI-2173, a Novel Liver-Targeted Non-Chain-Terminating Nucleotide for Hepatitis B Virus Cure Regimens
Source: Antimicrob Agents Chemother. 2020 Aug 20;64(9):e00836-20. doi: 10.1128/AAC.00836-20 (PMC7449170; doi:10.1128/AAC.00836-20)
Supplement: Supplemental file 1 [file AAC.00836-20-s0001.pdf]

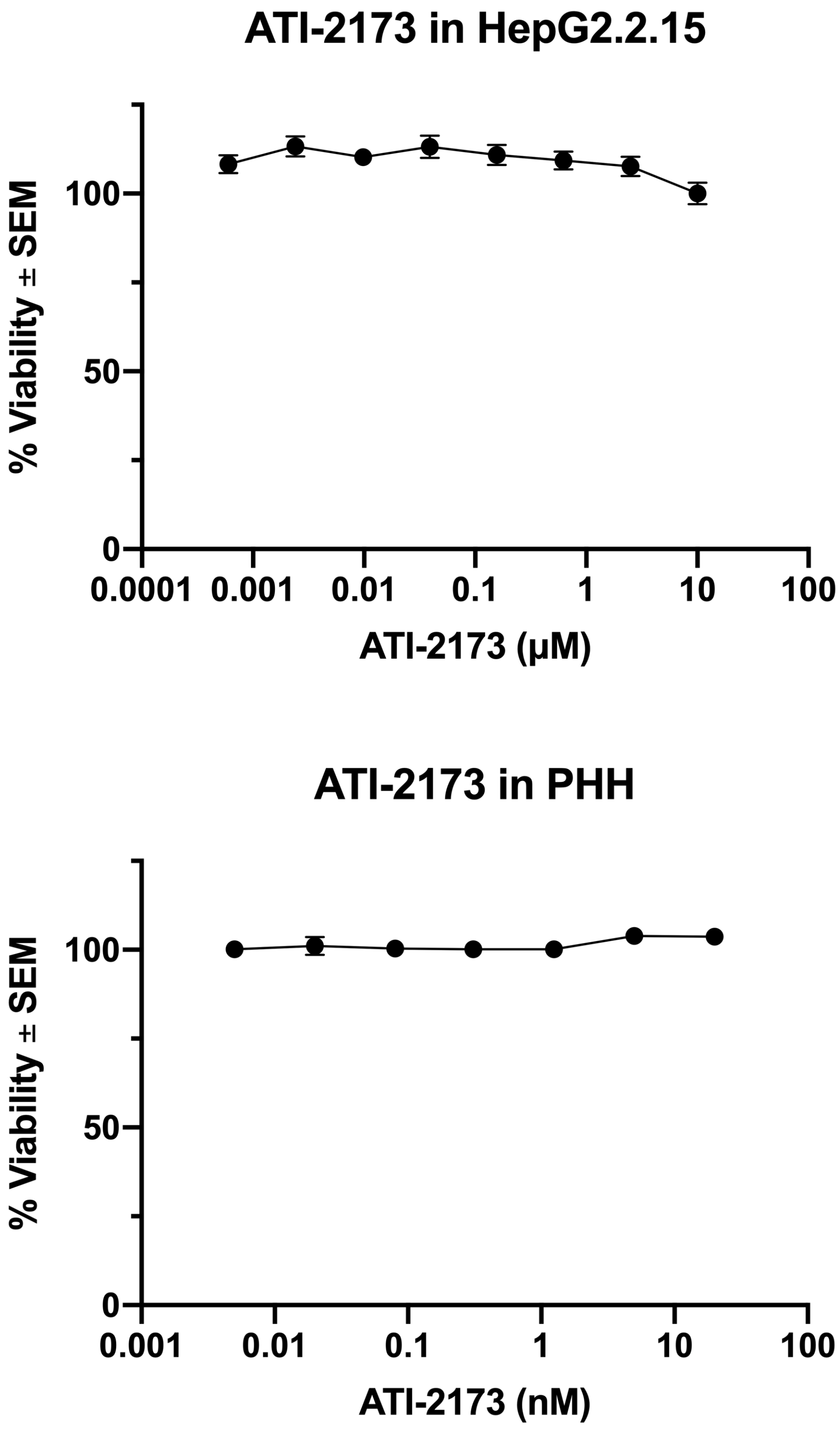


**Figure S1. Cell viability following ATI-2173 addition to HepG2.2.15 cells and primary human hepatocytes (PHH).**

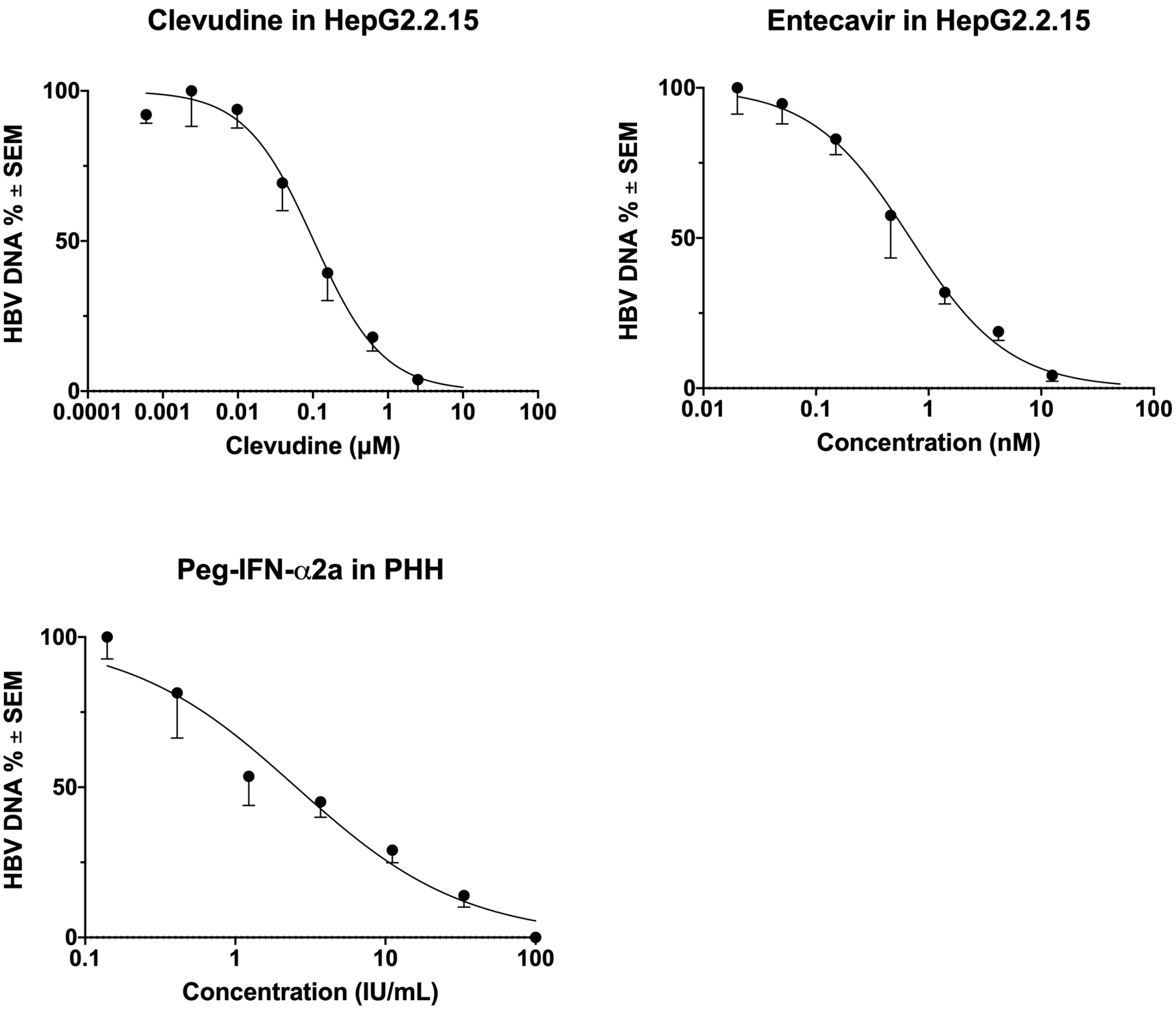


**Figure S2. Positive controls for anti-HBV activity assessments in HepG2.2.15 cells and primary human hepatocytes (PHH). Clevudine and entecavir were used as positive controls in HepG2 cells and inhibited HBV replication with $EC_{50}$s of 0.1μM and 0.6nM, respectively. Peg-interferon-α2a was used as a positive control in PHH and inhibited HBV replication with an $EC_{50}$ of 2 IU/mL.**

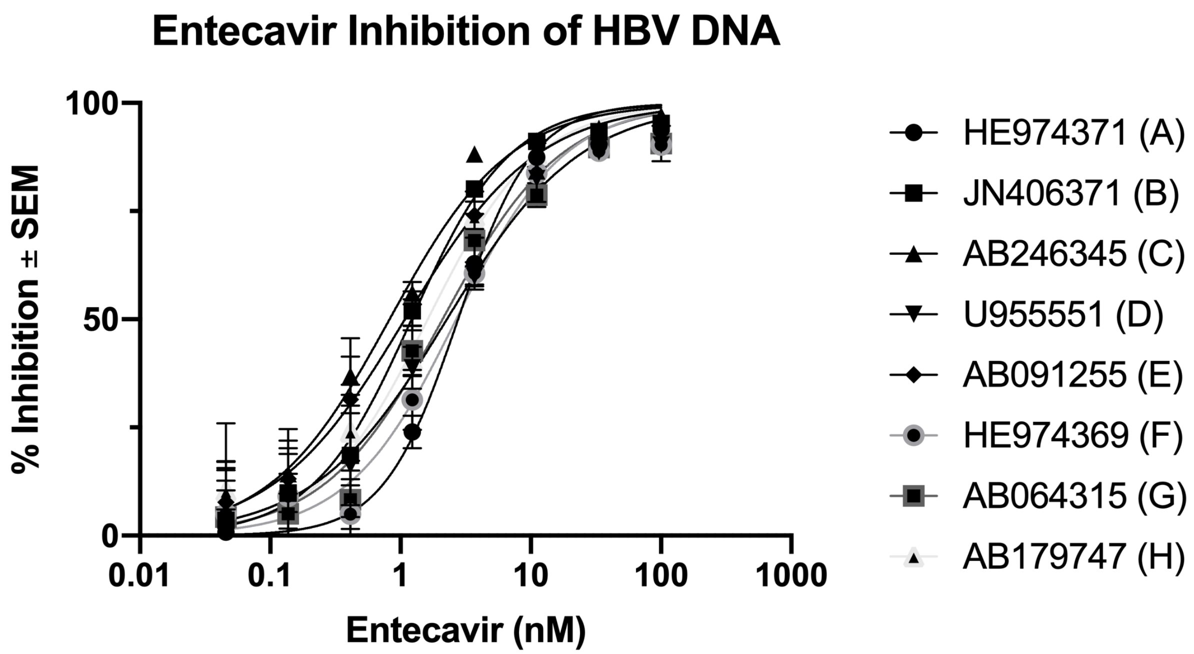


**Figure S3. Entecavir inhibition of HBV DNA across genotypes. $IC_{50}$s ranged from 0.5nM to 2.2nM. GenBank IDs correspond to the validated lab strains used in this assay.**

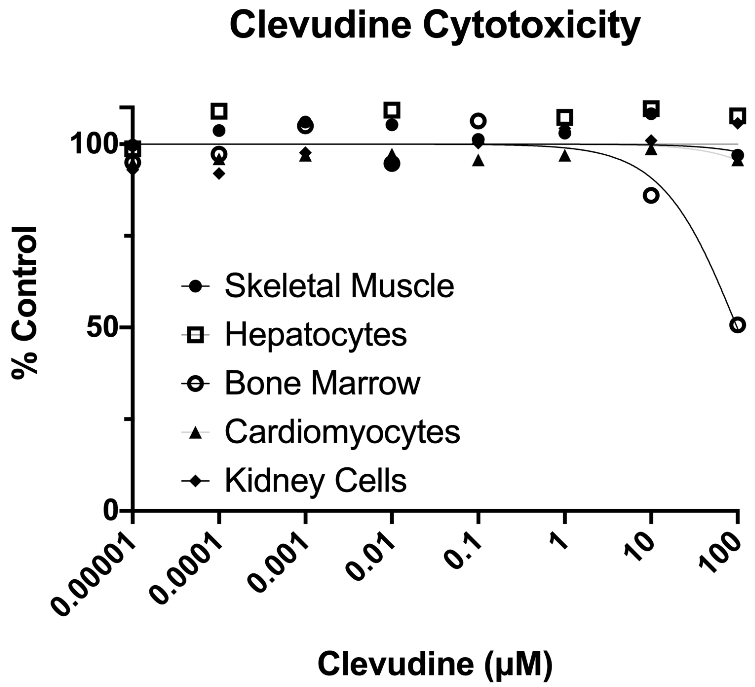


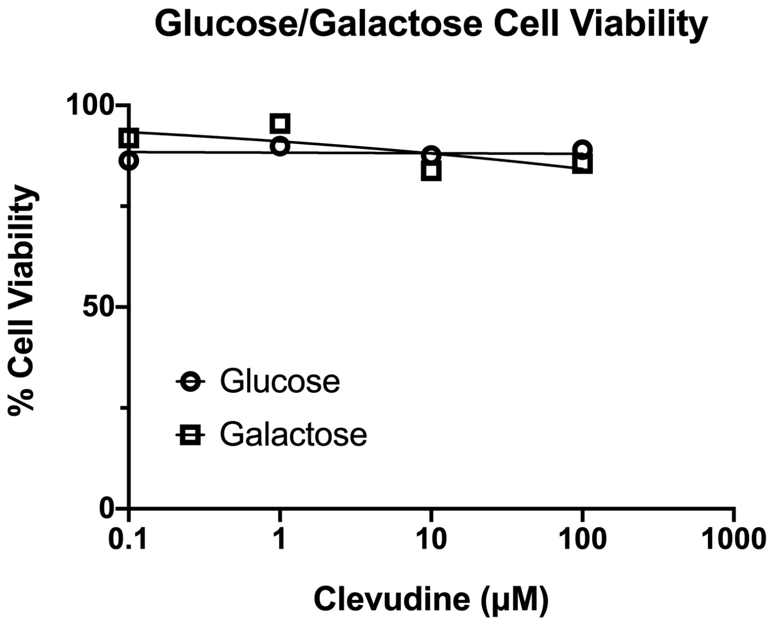


**Figure S4. Clevudine cytotoxicity and mitochondrial toxicity in multiple cell types. Clevudine was incubated with both proliferating and non-proliferating cells and was found to exhibit mild cytotoxicity in bone marrow cells with concentrations at or greater than 100µm. Clevudine was incubated in HepG2 cells in media supplemented with either 20mM glucose or 10mM galactose and showed no difference in cell viability in either media.**
